# Personal

**Published:** 1901-09

**Authors:** 


### PERSONAL.

DR. EUGENE SMITH, of Detroit, accompanied by Mrs. Smith, has been spending a few weeks at his country home on the Lakeshore, near Athol Springs. Dr. Smith and wife have also been occasional and interested visitors at the exposition grounds.

DR. LEWIS S. MCMURTRY and family, of Louisville, were visitors to Buffalo and the exposition for ten days in early August. They were charmed with the Fair.

DR. AND MRS. WILLIAM S. FOSTER, of Pittsburg, Pa., passed a few days in mid-August at the Kenilworth and were very greatly pleased with the exposition as well as with the attractiveness of Buffalo as a city.

DR. CHARLES A. L. REED and family, of Cincinnati, paid a ten-day visit to Buffalo in the early part of August and were greatly pleased with the exposition.

DR. ALTON L. SMILEY,, of Buffalo, U. of B., 1900, has been appointed junior physician at the Manhattan State Hospital, at a salary of $900.

DR. N. R. COLEMAN and wife of Columbus, O., spent a few days at the exposition during the latter days of August and spoke in glowing terms of its beauties, attractions and educational points of interest.

DR. AND MRS. GEORGE HENRY FOX, of New York, were among the late August visitors to Buffalo and the exposition. Dr. Fox accorded unstinted praise to the plan, scope and detail of the exposition.

MR. WILLIAM WHITFORD, of Chicago, the most accomplished medical stenographer in the country, paid a short visit to Buffalo and the exposition in the latter days of August. He expressed great interest and pleasure in what he saw.

